# Prevalence and risk factors for drug-induced cutaneous reactions among hospitalised patients: A retrospective study

**DOI:** 10.6026/973206300213461

**Published:** 2025-10-31

**Authors:** Vineet Kumar Sahu, Supriya Shakya, Anshuli Trivedi, Durgesh Sonare

**Affiliations:** 1 Department of Dermatology, Venereology & Leprosy, People's College of Medical Sciences & Research Centre, Bhopal, Madhya Pradesh, India; 2 Department of Dermatology, Venereology and Leprosy, NSC GMC, Khandwa, Madhya Pradesh, India; 3 Department of Community Medicine, Gandhi Medical College, Bhopal, Madhya Pradesh, India; 4 Department of Dermatology Venereology and Leprosy, NSC GMC, Khandwa, Madhya Pradesh, India

**Keywords:** Retrospective studies, polypharmacy, dermatological toxicity, hypersensitivity, hospitalized patients, medication-induced cutaneous reactions, adverse drug reactions, risk factors

## Abstract

One of the most frequent adverse medication events seen in hospitalized patients is drug-induced cutaneous response (DICR). Hence, 143 inpatients that experienced dermatological symptoms as a result of pharmaceutical treatment were assessed in this retrospective investigation. Maculopapular rashes were the most common appearance and the two main medication classes implicated were antibiotics and anticonvulsants. There were notable correlations seen with advanced age, polypharmacy and a history of medication allergies. Early detection of high-risk profiles can improve patient safety and pharmacovigilance.

## Background:

Drug-induced cutaneous responses (DICRs), which can vary from minor exanthematous eruptions to potentially fatal disorders like toxic epidermal necrolysis (TEN) and Stevens-Johnson syndrome (SJS), are common and frequently go unreported [1]. These skin reactions are some of the most obvious and recognizable signs of drug hypersensitivity and they frequently lead to stopping the offending substance [2]. Multiple medication exposure, underlying comorbidities and altered metabolic states make hospitalized patients especially vulnerable [3]. The pharmacological classes most frequently implicated are chemotherapeutic agents, nonsteroidal anti-inflammatory medicines (NSAIDs), antibiotics and anticonvulsants [4]. Risk factors that have been linked to an increased risk of acquiring DICRs include advanced age, female sex, polypharmacy, a history of drug allergies and renal or hepatic failure [5]. The exact prevalence and distribution of risk factors, however, may vary according on local prescribing patterns and demographic traits [6]. DICRs are frequently overlooked, particularly when they are mild, despite their possible clinical importance [7]. This mistake can raise the chance of development to more severe types and delay diagnosis [8]. Therefore, it is of interest to encourage early intervention and safer prescribing practices and it is imperative to have a better understanding of their prevalence and related risk factors in the hospital context. In addition to identifying the major clinical, pharmacological and demographic risk variables linked to the development of drug-induced cutaneous reactions, this retrospective study attempts to ascertain the prevalence of these reactions in hospitalized patients.

## Materials and Methods:

This 12-month retrospective analysis was carried out in a tertiary care facility. To find instances of drug-induced cutaneous responses among inpatients, the hospital's electronic medical records were examined. The study comprised 143 individuals who experienced new-onset skin lesions while in the hospital and who were identified as DICRs by an attending physician or dermatologist. Patients with pre-existing skin conditions, autoimmune dermatologic diseases, or uncertain etiology of rash were excluded. Demographic details including age, sex and comorbidities were recorded. Clinical details such as the type and duration of skin reaction, latency period between drug initiation and rash onset and severity (mild, moderate, severe) were documented. A detailed drug history was taken for each patient, focusing on recent medication exposure (within 1-3 weeks), total number of drugs received and identification of the most likely culprit agent based on the Naranjo adverse drug reaction probability scale. The implicated drugs were categorized into pharmacological classes (e.g., antibiotics, anticonvulsants, NSAIDs, etc.). Laboratory parameters including liver and renal function tests were also collected to evaluate systemic involvement. Data analysis was performed using statistical software. Prevalence was calculated as the proportion of patients who developed DICRs out of the total hospitalized population during the study period. Univariate and multivariate logistic regression analyses were used to identify independent risk factors associated with DICRs. A p-value <0.05 was considered statistically significant.

## Results:

A total of 143 cases of drug-induced cutaneous reactions (DICRs) were identified among 3,172 hospitalized patients, yielding a prevalence rate of 4.51%. The mean age of affected patients was 52.8 ± 16.3 years and there was a slight female predominance (54.5%). The most frequent type of reaction was maculopapular rash (46.9%), followed by urticaria (21.7%) and fixed drug eruptions (13.3%), with severe forms such as Stevens-Johnson syndrome and toxic epidermal necrolysis observed less frequently. Most reactions developed within 5-12 days of initiating the offending drug. Antibiotics were the leading cause (41.3%), with amoxicillin-clavulanate, ceftriaxone and ciprofloxacin most frequently implicated. Polypharmacy was a significant contributor, with nearly 63% of affected patients exposed to five or more drugs simultaneously. A prior history of drug allergy was reported in about one-third (32.9%) of cases, further increasing susceptibility. The majority of DICRs were mild to moderate in severity (91%), though 9.1% were classified as severe. Comorbidities were common, with hypertension (44%) and diabetes (40.6%) most prevalent among affected patients. Multivariate analysis identified age >60 years, female sex, polypharmacy and a history of drug allergy as significant independent risk factors for DICRs. Table 1 (see PDF) shows the demographic characteristics of patients with DICRs, including age distribution and gender, highlighting predominance in middle-aged and older individuals with a slight female preponderance. Table 2 (see PDF) presents the distribution of cutaneous reaction types, showing maculopapular rash as the most frequent form, followed by urticaria and fixed drug eruptions. Table 3 (see PDF) depicts the latency period for DICRs, demonstrating that most reactions occurred between 5 and 12 days after drug initiation. Table 4 (see PDF) shows the drug classes most frequently associated with DICRs, with antibiotics leading, followed by anticonvulsants and NSAIDs. Table 5 (see PDF) outlines the individual antibiotics implicated, with amoxicillin-clavulanate, ceftriaxone and ciprofloxacin accounting for the majority of antibiotic-related cases. Table 6 (see PDF) presents the relationship between drug exposure and DICRs, showing polypharmacy (≥5 drugs) as strongly associated with reactions. Table 7 (see PDF) summarizes the association of prior allergy history with DICRs, showing that nearly one-third of patients had a documented history of drug allergy. Table 8 (see PDF) displays the severity of DICRs, with most cases being mild to moderate and a smaller proportion categorized as severe. Table 9 (see PDF) lists systemic comorbidities among patients with DICRs, including hypertension, diabetes and chronic kidney disease. Table 10 (see PDF) shows the results of multivariate analysis, identifying age >60 years, female sex, polypharmacy and a history of drug allergy as significant independent predictors of DICRs.

## Discussion:

This retrospective study revealed a 4.51% prevalence of drug-induced cutaneous reactions (DICRs) among hospitalized patients, which is consistent with prior reports from similar tertiary care settings. The predominance of maculopapular rashes as the most frequent presentation supports existing literature, affirming its status as the most common morphological pattern of DICRs. The slightly higher prevalence in females may be attributed to sex-based immunological differences and increased healthcare-seeking behaviour [9]. Antibiotics-particularly beta-lactams like amoxicillin-clavulanate and cephalosporins-were the leading cause of cutaneous reactions. This is likely due to their widespread and sometimes empirical usage in hospitalized patients. Anticonvulsants such as phenytoin and carbamazepine were the second most frequent offenders, which aligns with their well-known hypersensitivity potential [10]. NSAIDs also featured prominently, further emphasizing the need for caution with these commonly prescribed medications. The majority of DICRs appeared 5-12 days after starting the medication, according to latency analysis, which is consistent with a delayed-type hypersensitivity mechanism. This emphasises how crucial it is to keep a tight eye on patients for the first two weeks of drug administration, particularly when using high-risk drugs [11]. The risks of a DICR were more than tripled when polypharmacy was found to be a significant independent predictor. The idea of reducing needless medicine and routinely evaluating drug regimens is supported by this finding, especially for older adults and people with several comorbidities. A major risk factor was also a history of pharmaceutical allergies, emphasising the significance of thorough allergy reporting and cautious drug selection in sensitive individuals [12]. Comorbidities such diabetes, hypertension and renal failure were frequently found in people with DICRs, despite the fact that their independent role as risk factors was not statistically confirmed in the multivariate model. Indirectly, these disorders may contribute to hypersensitivity by changing medication metabolism or immunological responses. Even though most DICRs were mild and self-limiting, a sizeable percentage (9.1%) were categorized as severe, which included disorders like SJS/TEN that could be fatal. This necessitates prompt identification and action to stop morbidity and death [13]. Prompt removal of the offend chemical and dermatological consultation continue to be the mainstays of therapy. The use of established diagnostic criteria and a clearly defined inpatient group are two of this study's advantages [14]. However, its retrospective designs, possible underreporting of mild instances and dependence on clinical judgment instead of challenge testing or biopsy for causality proof are some of its drawbacks. All things considered, this study reaffirms the clinical relevance of drug-induced cutaneous reactions in hospital environments and emphasizes the significance of focused preventive measures like reducing polypharmacy, identifying high-risk drugs and closely examining patient allergy histories. These actions are essential for enhancing pharmacovigilance procedures and patient safety.

## Conclusion:

The most common causes of drug-induced cutaneous responses, which affected 4.51% of hospitalized patients, were NSAIDs, antibiotics and anticonvulsants. Older age, female sex, polypharmacy and previous medication allergy were significant risk factors. To avoid serious consequences, better prescribing procedures and early detection are crucial.
